# A Challenging Case of Rapid Progressive Kaposi Sarcoma After Renal Transplantation: Diagnostics by FDG PET/CT

**DOI:** 10.1097/MD.0000000000000067

**Published:** 2014-08-29

**Authors:** Stefan Reuter, Alexis Vrachimis, Sebastian Huss, Eva Wardelmann, Mathias Weckesser, Hermann Pavenstädt

**Affiliations:** Department of Medicine D (SR, HP); Department of Nuclear Medicine (AV, MW); and Gerhard-Domagk-Institute of Pathology (SH, EW), University of Münster, Münster, Germany.

## Abstract

De-novo malignancy is a serious posttransplant complication. While the incidence of Kaposi sarcoma (KS) is low, the time for its diagnosis is early after renal transplantation. Typically, it can be identified because of the classical skin lesion. We herein report an unusual case of rapid progressive KS without skin lesions in a 52-year-old patient leading to death within 8 months after kidney transplantation. This striking case illustrates the usefulness of [^18^F]2-fluoro-2-deoxy-D-glucose positron emission tomography/computed tomography for demonstrating the cause of unexplained deterioration of patient’s condition. Early identification of KS is critical because early (modification of) therapy can substantially improve patient’s prognosis.

## INTRODUCTION

We herein report the exceptional case of a patient who died because of very early, disseminated Kaposi sarcoma (KS) without skin lesions after allogeneic kidney transplantation. The unusual course as well as the absence of cutaneous metastases led to a challenging diagnostic workup of the patient. Moreover, KS developed under an immunosuppressive regimen using mechanistic target of rapamycin (m-TOR) inhibition which is considered to be an effective treatment for KS. Ultimately, [^18^F]2-fluoro-2-deoxy-D-glucose (FDG) positron emission tomography/computed tomography (PET/CT) allowed diagnosis of disseminated malignancy and might therefore be considered early in the management of patient at risk.

## THE CASE

**FIGURE 1:**
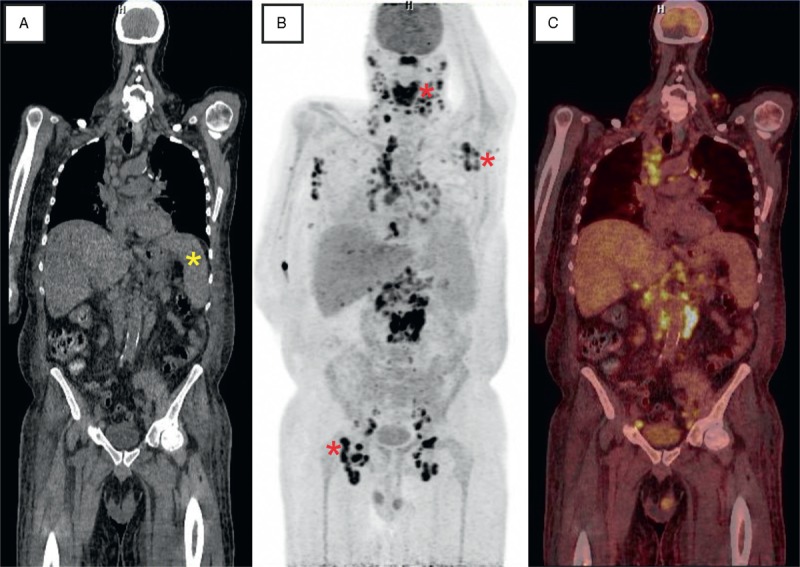
Slices of fluorodeoxyglucose PET combined with computed tomography (A: CT; B: PET; C: fusion of PET/CT). Besides a splenomegaly (yellow asterisk) a very intensive uptake was measured in nearly all lymph node stations. In particular, pathologic [^18^F]2-fluoro-2-deoxy-D-glucose accumulation was detected in cervical, axillary, mediastinal, paraaortic, and inguinal lymph nodes (red asterisks) as well as in the tongue, thyroid, and lung (MIP–PET). CT = computed tomography, MIP = maximum intensity projection, PET = positron emission tomography.

A 52-year-old white with end-stage renal failure secondary to rapid progressive glomerulonephritis presented 4 months after first renal transplantation with undulating fever, acute gastroenteritis, axillary abscesses, and a strong reduction of his general state. Laboratory results revealed thrombocytopenia (108 × 10^3^/μL, reference range 166–308 × 10^3^/μL), anemia (hemoglobin 87 g/L), acidosis, urinary tract infection with *Enterococcus faecalis*, as well as acute kidney injury. Immunosuppressive induction therapy included tacrolimus, everolimus, steroids, and basiliximab whereas maintenance treatment (ATHENA study protocol) consisted of tacrolimus (3–5 ng/mL), everolimus (3–8 ng/mL), and prednisolone 5 mg/day. Laboratory screening for cytomegalovirus, Epstein–Barr virus (EBV), hepatitis C virus, parvo B19, and human immunodeficiency virus was negative. Antibiotic therapy was commenced and the abscesses were surgically drained. The patient’s general state improved, he was without fever, and thrombocytes and hemoglobin increased while creatinine decreased. Two months after discharge, the patient was hospitalized because of acute gastroenteritis, sepsis, and acute kidney injury again. On physical examination, no suspicious skin lesions or lymph nodes were noted. Laboratory analyses showed increased procalcitonin (2.9 ng/mL, reference range <0.5 ng/mL), C-reactive protein (10.5 mg/dL, reference range <0.5 mg/dL), creatinine (2.2 mg/dL, baseline creatinine 1.5 mg/dL), and lactate dehydrogenase (328 U/L, reference range 135–255 U/L). Blood differential count revealed anemia (hemoglobin 80 g/L), thrombocytopenia (60 × 10^3^/μL), mild lymphocytopenia (0.95 × 10^3^/μL, reference range 1.26–3.35 × 10^3^/μL), and monocytosis (1.84 × 10^3^/μL, reference range 0.29–0.95 × 10^3^/μL). *Staphylococcus epidermidis* was isolated from multiple blood cultures and from the relapsed axillary abscess (shown by asterisk in Figure 1B). Moreover, EBV polymerase chain reaction (PCR) testing was slightly positive (354 IU/mL) and *Clostridium difficile* was identified in the stool. Sonography revealed splenomegaly (18 × 7.6 cm, also seen in CT, shown by asterisk in Figure 1A) and cervical as well as reactive inguinal lymph nodes (≤2.3 cm). Treatment included fluid replacement and antibiotics. As the patient complained of progressive intolerance of everolimus, immunosuppressive therapy was modified (everolimus was stopped and tacrolimus was reduced). Persisting fever and coughing led to the performance of CT of the thorax excluding everolimus-induced pneumonitis and showing pulmonary emphysema and multiple enlarged but calcified mediastinal and hilar lymph nodes. Interestingly, the patient’s condition improved, but persisting thrombocytopenia and anemia led us to puncture and biopsy the bone marrow (iliac crest biopsy). Toxic or infectious bone marrow suppression as well as folic acid deficiency was suspected. Later, the patient developed dysphagia due to a bleeding tongue ulceration. The patient recovered slowly and was discharged. Two weeks later, the patient developed fever, massive thrombocytopenia (12 × 10^3^/μL), and acute kidney failure. Further diagnostics included combined PET/CT with FDG. Besides of the very intensive uptake measured in nearly all lymph node stations, in particular cervical, axillary, mediastinal, paraaortic, and inguinal, an pathological uptake was documented in the tongue, thyroid, and lung (Figure 1B and C); the uptake pattern was indicative for malignancy (coronal slice of CT, maximum intensity projection (MIP)–PET, and fused coronal slice of FDG–PET/CT). Extirpation of an inguinal lymph node (shown by asterisk in Figure 1B) revealed fast proliferating KS. The patient died before palliative chemotherapy with doxorubicin could be started.

## DISCUSSION

**FIGURE 2:**
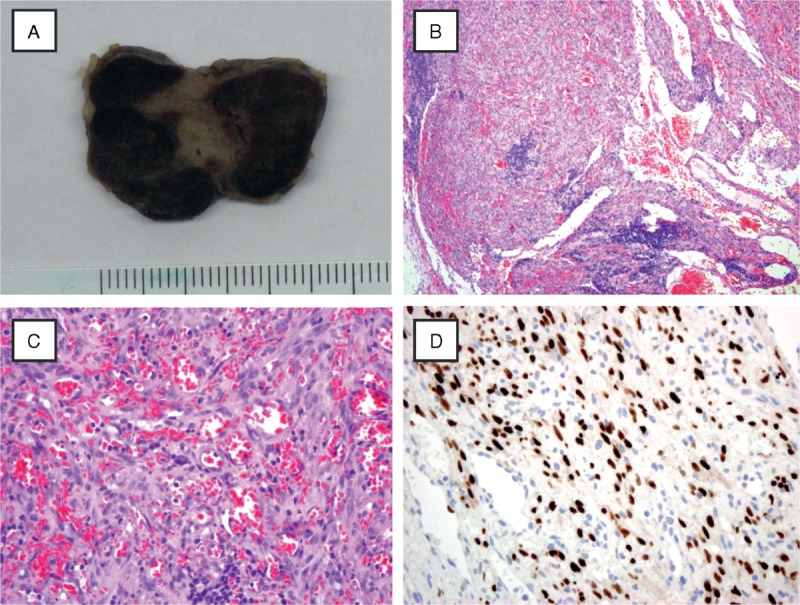
Histological evaluation of an inguinal lymph node (gross specimen, A) revealed only residual lymphatic tissue (low power 10×, B) and showed a diffuse endothelial neoplasm with scattered lymphocytes and plasma cells. Mild cytologic atypia was noted (high power 40×, C). Immunohistochemistry for HHV-8 showed intense HHV-8 nuclear positivity and the final diagnosis of KS was established (40×, D). HHV-8 = human herpesvirus-8, KS = Kaposi sarcoma.

Although extremely rare (incidence below 1% within 15 years after renal transplantation), KS has been described to occur early (mean time to diagnosis: 426 days after renal transplantation).^1,2^ KS is a vascular low-grade malignant tumor that is associated with human herpesvirus-8 (HHV-8) infection.^3^ Interestingly, in our patient, HHV-8 staining of the lymph node was positive, whereas serum PCR was negative (Figure 2B). It typically manifests in mucocutaneous sites such as the skin or the oropharyngeal mucosa, in lymph nodes, and in visceral organs, most frequently in the respiratory and gastrointestinal tract. In our patient, typical, for example, lymph nodes, and atypical manifestations, for example, thyroid, were seen (only 5 cases worldwide).^4^ In the absence of skin lesions (only 5% of cases and exceptional in metastatic disease), KS often proves to be a challenging diagnosis because of missed recognition on routine imaging studies, unspecific systemic manifestations, for example, anemia, thrombocytopenia, and fever, or infectious complications, for example, abscesses, diarrhea, EBV reactivation, urinary tract infection, or sepsis, especially in the setting of our immunocompromised host. Awareness of KS may avoid delayed or potential misdiagnosis with deleterious consequences. In our patient, the combination of HHV-8 and immunosuppression promoted the development of KS. Notably, KS occurred under maintenance therapy with an m-TOR inhibitor (everolimus). m-TOR inhibition is considered to be effective for the treatment of KS because it is antiangiogenetic by reduction of vascular endothelial growth factor secretion and inhibition of formation of tumor vasculature.^5^

KS can be clearly visualized as areas of intense FDG uptake on a PET scan (Figure 1B). As bone marrow biopsy specimens were unspecific in KS, unexplained cytopenias should lead to further work-up.^6^ Thus, FDG–PET imaging can be advantageous in the management of patients with suspicious malignancy or unclear inflammatory/infectious diseases.^7^ FDG–PET provides noninvasive whole-body diagnostics considerably assessing the extent of the disease (useful for both staging and restaging), that is, for the detection of further sites involved yet uncovered by conventional diagnostic methods. It might, therefore, be considered early in the management of patient at risk to reveal underlying disease (for timeline of the diagnostic workup and for key findings, see Table 1). Herein, combined FDG PET/CT ultimately allowed diagnosis of disseminated malignancy. However, because of the late stage of KS on the time of diagnosis, it was too late for initiation of chemotherapy.

**TABLE 1:**
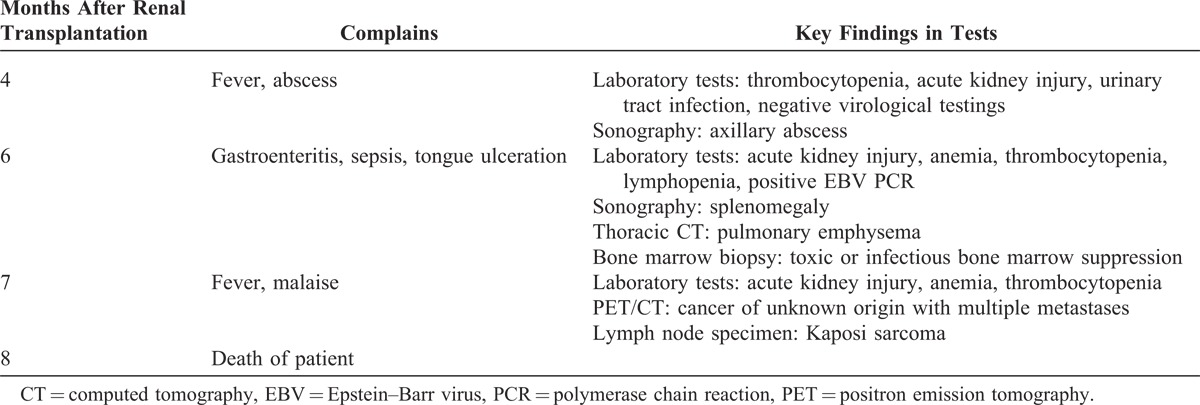
Timeline of the Diagnostic Workup
